# Supplementary material for: Improving health, well-being and parenting skills in parents of children with medical complexity: a scoping review protocol
Source: BMJ Open. 2017 Sep 24;7(9):e015242. doi: 10.1136/bmjopen-2016-015242 (PMC5623464; doi:10.1136/bmjopen-2016-015242)
Supplement: Author's manuscript [file bmjopen-2016-015242.draft_revisions.pdf]

BMJ Open is committed to open peer review. As part of this commitment we make the peer review history of every article we publish publicly available.

When an article is published we post the peer reviewers' comments and the authors' responses online. We also post the versions of the paper that were used during peer review. These are the versions that the peer review comments apply to.

The versions of the paper that follow are the versions that were submitted during the peer review process. They are not the versions of record or the final published versions. They should not be cited or distributed as the published version of this manuscript.

BMJ Open is an open access journal and the full, final, typeset and author-corrected version of record of the manuscript is available on our site with no access controls, subscription charges or pay-per-view fees (http://bmjopen.bmj.com).

If you have any questions on BMJ Open's open peer review process please email editorial.bmjopen@bmj.com

BMJ Open

Improving health, wellbeing and parenting skills in parents of children with medical complexity- A scoping review protocol

Journal:	*BMJ Open*
Manuscript ID	bmjopen-2016-015242
Article Type:	Protocol
Date Submitted by the Author:	22-Nov-2016
Complete List of Authors:	Bradshaw, Sally; University of Birmingham, Institute of Applied Health Research<br>Shaw, Karen; University of Birmingham<br>Bem, Danai; University of Birmingham, Institute of Applied Health Research<br>Cummins, Carole; University of Birmingham, Institute of Applied Health Research
**Primary Subject Heading**:	Public health
Secondary Subject Heading:	Paediatrics, Evidence based practice
Keywords:	scoping review, children, parents, chronic illness, interventions

SCHOLARONE™ Manuscripts

For peer review only

Improving health, wellbeing and parenting skills in parents of children with medical complexity- A scoping review protocol

First author: Sally Bradshaw

Co-authors: Karen Shaw, Danai Bem, Carole Cummins

Corresponding author contact details:

Sally Bradshaw
Institute of Applied Health Research
College of Medical and Dental Sciences
Room 136, Murray Learning Centre
University of Birmingham
Edgbaston
Birmingham B15 2TT
Tel: 0121 414 8374
Fax: N/A
Email: s.r.bradshaw@bham.ac.uk

Co-author details:
Karen Shaw
Institute of Applied Health Research
College of Medical and Dental Sciences
University of Birmingham
Birmingham

Danai Bem
Institute of Applied Health Research
College of Medical and Dental Sciences
University of Birmingham
Birmingham

Carole Cummins
Institute of Applied Health Research
College of Medical and Dental Sciences
University of Birmingham
Birmingham

Keywords or phrases suitable for use in an index
scoping review, children, parents, chronic illness, interventions

Word count - excluding title page, references, figures and tables.
2315

For peer review only

Abstract

Introduction: Less than 1% of children have complex medical conditions but account for one third of all child health spending. The impact of suboptimal management of this group of children can have a considerable effect on families as well as services. Some families appear to cope more easily than others do, but there are compelling reasons to suggest that effective interventions may improve family coping and ultimately outcomes. Hospitalisation of their child presents a unique set of pressures and challenges for parents, but also an opportunity to intervene. However, the evidence is not well described in relation to this group of families. The primary objective of this scoping review is to identify interventions available to improve parent-child attachment, parental health, wellbeing, functioning or skills in this context.

Methods and analysis: Nine bibliographic databases will be searched spanning medicine, nursing, psychology, education, social work and the grey literature using a combination of index terms and text words related to parents, childhood, chronic illness and interventions. Study eligibility will be assessed by two researchers against pre-set inclusion and exclusion criteria. Key information from each study will be extracted and charted including year of publication, condition, severity, geographical setting, key concepts and definitions, aims, study population and sample size, methodology / methods, interventions, outcomes, and key findings. Directed qualitative content analysis will be used to make sense of narrative findings within the included studies. Results will be presented which summarise the scope of the literature and identify key findings, potential areas for evidence synthesis and research gaps.

Ethics and dissemination: Ethical approval is not required. The results of this review will be disseminated through publication in a peer-reviewed journal and feedback to stakeholders during the development of a hospital based intervention.

Abstract word count: 289

Strengths and limitations of this study

- This review will describe the literature in relation to a key opportunity to intervene to improve outcomes
- This review will be based on a robust definition of children with medical complexity
- Formal scoping review methodology will be used to provide a systematic, rigorous, transparent and reproducible review
- This will not be a systematic review but will identify focussed areas for systematic review

For peer review only

For peer review only - http://bmjopen.bmj.com/site/about/guidelines.xhtml

Background

This scoping review has been designed to inform development of an intervention to support parents of children with medical complexity around the time of hospital admission. It has been estimated that whilst children with the most complex medical needs include less than 1% of the child population they account for one third of all child health spending.[1] The impact of suboptimal management of this group of children, which can include the support given to enable successful family adjustment as well as medical management, can have a considerable effect on families as well as child health services and budgets.[2]

There is a well-established definition for children with special health care needs (CSHCN) which encompasses those children who have or are at increased risk of a chronic physical, developmental, behavioural, or emotional condition and require health care and related services of a type or amount beyond that required by children generally.[3] Definitions for groups of children with the most severe chronic diseases or diseases with the most serious long-term effects are less well established. We have adopted the definition of 'children with medical complexity' developed by Cohen [2] which is based on a systematic review of definitions of childhood chronic conditions.[4] Cohen's definitional framework includes four domains:

- Substantial family identified service needs and / or significant impact on the family (e.g. financial burden)
- Diagnosed or undiagnosed chronic condition which is severe or associated with medical fragility
- Severe functional limitations and / or dependence on technology
- High health care use and / or engagement with multiple service providers that may include non-medical providers.

When describing the wider population of children with any chronic health condition we will use the term 'children with special health care needs (CSHCN)'.[3] When referencing source literature, the original terminology will be used—e.g. chronic conditions—in order to retain a sense of the original meaning.

Most parents adjust to their child's illness successfully. [5 6] However not all families do adjust well and poor adjustment has been associated with poorer health outcomes for parents, the ill child and other family members. [7] A recent meta-analysis of 37 studies where the relationship between family functioning and child wellbeing in children with chronic health conditions were analysed found significant correlations between family functioning and children's problem behaviours, social competence, quality of life, medication adherence and physical health. [8]

For peer review only - http://bmjopen.bmj.com/site/about/guidelines.xhtml

Whilst some families appear to cope more easily than others there are compelling reasons to suggest that effective interventions may improve outcomes for parents and their families. Some factors that predict adjustment may not be particularly open to hospital based intervention such as family environment, illness severity and chronicity (the long-term nature of the diagnosis). [9] However other factors that have been identified as facilitators of successful adjustment are more amenable to hospital based intervention. These include focussing on the child's achievements; performing care routines; becoming flexible in relation to care and treatment routines; developing knowledge of the condition and treatments; being able to learn from illness episodes and apply that learning to future situations; and developing effective relationships with staff. [5]

Several authors highlight the importance of the illness trajectory. Burden [10] suggests that there are opportunities for professionals to support parents to successfully adjust to their child's diagnosis. Rolland and Walsh identify three major phases of childhood chronic disease: crisis (pre-diagnosis and initial adjustment); chronic (the long haul); and terminal phases in progressive conditions. [11] These phases pose distinct challenges and are likely to be associated with healthcare contact and opportunities for supportive interventions to promote resilience and adjustment. The potential benefits of parenting programmes are highlighted in the findings of two reviews. A Cochrane review of group-based parenting interventions to improve parental psycho-social health found evidence to support the use of parenting programmes [12] and a separate Cochrane review found some evidence that psychological therapies are beneficial for parents of children with special health care need.[13] Further evidence covering related issues have also been reviewed, for example research on improving or supporting professional-parent collaborations in managing children with special health care needs,[5 14] nursing research on parenting children with complex chronic conditions,[15] the nature of family engagement in interventions for this population,[16] and the role of interactive media for parental education.[17]

However, whilst these reviews provide valuable insights they do not provide a comprehensive evidence base for the context of children with medical complexity around the point of hospitalisation. Much of the available review evidence only addresses pre-determined categories of interventions, (e.g. group [10], psychological [11], media [15]), and do not address other potentially important parent and family support functions such as social support, chronic illness education and skill development, or support with relevant common parenting issues. In addition, they are not always well tailored to the specific parenting challenges around children with medical complexity. This review will address this knowledge gap by scoping a broad range of interventions that have been tested within populations of children with special health care needs (CSHCN). This is important

For peer review only - http://bmjopen.bmj.com/site/about/guidelines.xhtml

because there is likely to be a broad range of relevant evidence that has not yet been scoped, and this will allow that evidence to be identified, characterised, and assessed in relation to the needs of parents of children with medical complexity.

Methods / design

Research question and objectives

The research questions for this review are (1) What interventions are available to improve health, wellbeing, functioning or skills in parents of children with special health care needs (CSHCN), and do they work? (2) Who are the study populations, what were the intervention targets, and which outcomes have been measured? (3) To what extent are the results relevant and transferable to delivery around the time of routine health care contact?

Study design

Formal scoping review methodology will be used [18-22]. This approach has been chosen to provide a rigorous, transparent and reproducible method for scoping a research area that includes a systematic search strategy and data extraction, but will not exclude studies based on design or quality. This allows the full extent of the relevant literature to be included and described, which is useful where an area is complex or has not been comprehensively reviewed before.[19]

Eligibility criteria

The Population, Intervention, Comparator, Outcome (PICO) framework has been used to define the review focus and a PICO statement can be found in Table 1.

Table 1: PICO Statement

Population	Parent of CSHCN
Intervention	Any parent or family based intervention
Comparator	Usual care or any other comparator
Outcome	Improved parenting health, wellbeing, functioning or skills

Detailed study eligibility criteria can be found in Table 2.

Table 2: Inclusion and Exclusion Criteria

For peer review only - http://bmjopen.bmj.com/site/about/guidelines.xhtml

**Inclusion criteria**	
Types of studies	Any reports of interventions using a recognised study design (including primary or secondary research).<br><br>Interventions must aim to improve health and wellbeing, functioning or skills in parents of children with special health care needs.
Setting	Studies undertaken in any research setting (e.g. acute, primary care, community) will be included, as long as the intervention could potentially be delivered within routine care in an acute setting by existing health staff, peer-supporters or volunteers.
Population	'Parent' may include anyone with parenting responsibility.<br><br>Children with special health care needs (CSHCN): children who have or are at increased risk of a chronic physical, developmental, behavioural, or emotional condition and require health care and related services of a type or amount beyond that required by children generally.
Intervention	Interventions must include parents directly. They may include only parents, or parents alongside children and / or other family members.<br><br>Interventions may include but are not limited to peer-support, listening and encouraging, education, training, enablement, modelling, or environmental restructuring (for example care environments). Single disease studies will be included (for example cardiac conditions, cancer, metabolic conditions) as long as they meet the above inclusion criteria, as well as studies that include parents of children with a variety of clinical conditions.
Outcomes	Improved parent-child attachment or parenting health, wellbeing, functioning or skills.<br><br>'Health and wellbeing' may include patient reported outcome measures

For peer review only - http://bmjopen.bmj.com/site/about/guidelines.xhtml

For peer review only

	(PROMs), happiness, psychological adjustment or adaptation, quality of life, resilience, coping or self-efficacy. It may also include reduction in negative outcomes including stress, anxiety, depression, or physical health measures.<br><br>Parental functioning and skills refers to a range of parenting behaviours including nurturing, discipline, teaching, monitoring, and management. [24]
**Exclusion criteria**	

- Any studies which do not report parent outcomes.
- Interventions that are not adaptable to delivery by generalist healthcare staff or lay workers (e.g. specialist psychotherapy techniques).
- Studies which use parent- based interventions but only measure child wellbeing or disease related outcomes such as medication adherence.
- Studies that focus on acute conditions only (e.g. acute pneumonia).
- Studies that focus only on end of life care.
- Studies where the parent has the long-term condition.
- Studies on Attention Deficit Hyperactivity Disorder (ADHD), autism, depression or other mental health conditions in the absence of co-morbidities.

Search strategy

A comprehensive search strategy has been developed to identify both published and unpublished literature. It has been designed and will be performed with advice and support from a specialist in systematic reviews. A range of sources will be searched including the following disciplines: medicine, nursing, allied health professions, sociology, psychology, education and social work. Peer-reviewed, published and grey literature will be searched. Primary research studies that evaluate interventions using any methodology and secondary research studies including scoping reviews, systematic reviews and meta-analyses will be included.

Relevant studies will be identified through individual searches of relevant data bases. These will include Medline, Embase, PsychINFO, the Cochrane library, the Cumulative Index of Nursing and Allied Health Literature (CINAHL), Education Resources Information Centre (ERIC), and Applied Social Sciences Index and Abstracts (ASSIA). HMIC and OpenGrey will be searched for grey literature. Reference lists will be mined for additional references. No previous similar reviews have been found

For peer review only - http://bmjopen.bmj.com/site/about/guidelines.xhtml

and therefore no date restrictions will be applied. Searches will be restricted to English language papers.

A phased search strategy will be used. An initial search of Medline and CINAHL will be performed using the text words shown in Table 3 and related index terms.

Table 3 – Key word search terms

Key concept	Key words
Parents	parent, mother, father, carer
Child	child, school child, kid, toddler, teen, boy, girl, minor, underage, juvenile, youth, puberty, pubescent, prepubescent, pediatric, paediatric , school
Chronic childhood disease	Chronic, long term, activity limiting, disease, illness, disorder, condition, sickness, pain
Interventions to improve wellbeing	Intervention, therapy, trial, review

The primary researcher will screen initial search results, abstracts of relevant studies will be retrieved, and will be analysed by the same researcher for text-words contained in the titles and abstracts, as well as index terms used to describe the articles. In discussion with a systematic review specialist, the results from these first stage searches will be used to optimise the search strategy for second stage searching. The second stage search will be performed individually across all databases using all identified text words and index terms found in phase 1, with search terms and strategies optimised for each database.

Study selection

EndNote (Thomson Reuters, New York) will be used to manage the records identified from the literature search and to record decisions during the study selection process. Two researchers will screen all titles from the full search results and a third researcher will take a final decision where disagreements cannot be resolved. Full texts of all potentially relevant studies will then be retrieved in full and assessed by two researchers for a final inclusion decision. Finally, reference list mining will be used to identify any further eligible studies. The selection process will be illustrated using a Preferred Reporting Items for Systematic Reviews and Meta-Analyses flow diagram.

For peer review only - http://bmjopen.bmj.com/site/about/guidelines.xhtml

For peer review only

Data Analysis and synthesis

One researcher will extract data using a pre-specified data extraction form which will reflect the research questions, and this will be checked by a second researcher. Key information from each included study will be charted in a table which will include the author, year of publication, medical condition(s), severity, geographical setting, academic / professional discipline, key concepts and definitions, aims, study population and sample size, methodology / methods, intervention, outcomes, and key findings related to the research questions. This list is indicative only and the charting process will be iterative. As the reviewers become familiar with the evidence, the data extraction form may be updated with other headings to ensure that all relevant information is included.

In addition directed qualitative content analysis [23] will be undertaken to analyse any narrative data in included study reports. This approach to qualitative data analysis will allow exploration of narrative texts to focus on the review questions. Data will be coded according to predetermined codes derived from the research questions. Data that cannot be coded using these codes will be identified and analysed to determine whether they represent a new category or a subcategory of an existing code.

Presentation of results, discussion and conclusions

Results will be presented visually and descriptively. Key data will be presented in tables, including a main table of all interventions that meet the inclusion criteria. Additional data tables will summarise other key features including research methodology and design, study dates, medical condition(s), severity, geographical location, academic/ professional origin and intervention function. Results of the directed qualitative content analysis will accompany the tables to further explore and discuss key findings in relation to the scoping review questions and their implications. The discussion and conclusions will also address potential areas for evidence synthesis and any identified research gaps.

What this study will add

This study will describe the evidence base available for parenting interventions for parents of children with special health care need, and support development of interventions for children with medical complexity. This scoping review will contribute to a novel parent support intervention that can be delivered from within the hospital setting.

For peer review only - http://bmjopen.bmj.com/site/about/guidelines.xhtml

For peer review only

Ethics and dissemination

Scoping reviews do not require ethical approval. This protocol has not been prospectively registered on the PROSPERO database because scoping reviews are currently excluded. The results of this review will be disseminated through publication in a peer-reviewed journal and feedback to stakeholders during the development of the proposed hospital based intervention.

Authors' contributions

SB conceived of and wrote the protocol. CC and KS provided academic supervision and contributed to the final draft. DB provided detailed technical advice and contributed to the final draft.

Funding statement

Sally Bradshaw is funded by the NIHR CLAHRC West Midlands initiative. This paper presents independent research and the views expressed are those of the author(s) and not necessarily those of the NHS, the NIHR or the Department of Health.

Competing interests

None

References

1. Cohen E, Berry JG, Camacho X, et al. Patterns and costs of health care use of children with medical complexity. Pediatrics 2012;**130**(6):e1463-70.

2. Cohen E, Kuo DZ, Agrawal R, et al. Children with medical complexity: an emerging population for clinical and research initiatives. Pediatrics 2011;**127**(3):529-38.

3. McPherson M, Arango P, Fox H, et al. A New Definition of Children With Special Health Care Needs. Pediatrics 1998;**102**:137-40.

4. van der Lee J, Mokkink L, Grootenhuis M, et al. Definitions and Measurement of Chronic Health Conditions in Childhood A Systematic Review. Journal of the American Medical Association 2007;**297**(24):2741-51.

5. Smith J, Swallow V, Coyne I. Involving parents in managing their child's long-term condition-a concept synthesis of family-centered care and partnership-in-care. J Pediatr Nurs 2015;**30**(1):143-59.

For peer review only - http://bmjopen.bmj.com/site/about/guidelines.xhtml

For peer review only

6. Pinquart M. Do the parent-child relationship and parenting behaviors differ between families with a child with and without chronic illness? A meta-analysis. J Pediatr Psychol 2013;**38**(7):708-21.

7. Cousino MK, Hazen RA. Parenting stress among caregivers of children with chronic illness: a systematic review. J Pediatr Psychol 2013;**38**(8):809-28.

8. Leeman J, Crandell JL, Lee A, et al. Family Functioning and the Well-Being of Children With Chronic Conditions: A Meta-Analysis. Research in Nursing & Health 2016;**39**(4):229-43.

9. Morawska A, Calam R, Fraser J. Parenting interventions for childhood chronic illness: a review and recommendations for intervention design and delivery. J Child Health Care 2014;**19**(1):5-17.

10. Burden R. Psycho-social transitions in the lives of parents of children with handicapping conditions. Counselling Psychology Quarterly 1991;**4**(4):331-43.

11. Rolland JS, Walsh F. Facilitating family resilience with childhood illness and disability. Current Opinion in Pediatrics 2006;**18**:527-38.

12. Barlow J, Smailagic N, Huband N, et al. Group-based parent training programmes for improving parental psychosocial health (Review). The Cochrane Library 2012;**6**(Supp 2):1-205.

13. Eccleston C, Fisher E, Law E, et al. Psychological interventions for parents of children and adolescents with chronic illness. 2015.

14. Nightingale R, Friedl S, Swallow V. Parents' learning needs and preferences when sharing management of their child's long-term/chronic condition: A systematic review. Patient Educ Couns 2015;**98**(11):1329-38.

15. Rehm RS. Nursing's contribution to research about parenting children with complex chronic conditions: An integrative review, 2002 to 2012. Nursing Outlook 2013;**61**(5):266-90.

16. Knafl KA, Havill NL, Leeman J, et al. The Nature of Family Engagement in Interventions for Children With Chronic Conditions. West J Nurs Res 2016.

17. Annaim A, Lassiter M, Viera AJ, et al. Interactive media for parental education on managing children chronic condition: a systematic review of the literature. BMC Pediatrics 2015;**15**:1-13.

18. JBI. Joanna Briggs Institute Reviewers' Manual: 2015 edition/supplement. Adelaide: Joanna Briggs Institute, 2015.

19. Arksey H, O'Malley L. Scoping studies: towards a methodological framework. International Journal of Social Research Methodology 2005;**8**(1):19-32.

20. Davis K, Drey N, Gould D. What are scoping studies? A review of the nursing literature. Int J Nurs Stud 2009;**46**(10):1386-400.

For peer review only

For peer review only - http://bmjopen.bmj.com/site/about/guidelines.xhtml

21. Levac D, Colquhoun HL, O'Brien BC. Scoping studies: advancing the methodology. Implement Science 2010;**5**:69.

22. Colquhoun HL, Levac D, O'Brien KK, et al. Scoping reviews: time for clarity in definition, methods, and reporting. J Clin Epidemiol 2014;**67**(12):1291-4.

23. Hsieh HF, Shannon SE. Three approaches to qualitative content analysis. Qual Health Res 2005;**15**(9):1277-88.

For peer review only

For peer review only - http://bmjopen.bmj.com/site/about/guidelines.xhtml

BMJ Open

Improving health, wellbeing and parenting skills in parents of children with medical complexity- A scoping review protocol

Journal:	*BMJ Open*
Manuscript ID	bmjopen-2016-015242.R1
Article Type:	Protocol
Date Submitted by the Author:	20-Jun-2017
Complete List of Authors:	Bradshaw, Sally; University of Birmingham, Institute of Applied Health Research<br>Shaw, Karen; University of Birmingham<br>Bem, Danai; University of Birmingham, Institute of Applied Health Research<br>Cummins, Carole; University of Birmingham, Institute of Applied Health Research
**Primary Subject Heading**:	Public health
Secondary Subject Heading:	Paediatrics, Evidence based practice
Keywords:	scoping review, children, parents, chronic illness, interventions

SCHOLARONE™ Manuscripts

For peer review only

Improving health, wellbeing and parenting skills in parents of children with medical complexity- A scoping review protocol

First author: Sally Bradshaw

Co-authors: Karen Shaw, Danai Bem, Carole Cummins

Corresponding author contact details:

Sally Bradshaw
Institute of Applied Health Research
College of Medical and Dental Sciences
Room 136, Murray Learning Centre
University of Birmingham
Edgbaston
Birmingham B15 2TT
Tel: 0121 414 8374
Fax: N/A
Email: s.r.bradshaw@bham.ac.uk

Co-author details:
Karen Shaw
Institute of Applied Health Research
College of Medical and Dental Sciences
University of Birmingham
Birmingham

Danai Bem
Institute of Applied Health Research
College of Medical and Dental Sciences
University of Birmingham
Birmingham

Carole Cummins
Institute of Applied Health Research
College of Medical and Dental Sciences
University of Birmingham
Birmingham

Keywords or phrases suitable for use in an index
scoping review, children, parents, chronic illness, interventions

Word count - excluding title page, references, figures and tables.
2594

For peer review only

For peer review only - http://bmjopen.bmj.com/site/about/guidelines.xhtml

Abstract

Introduction: Less than 1% of children have complex medical conditions but account for one third of all child health spending. The impact of suboptimal management of this group of children can have a considerable effect on families as well as services. Some families appear to cope more easily than others do, but there are compelling reasons to suggest that effective interventions may improve family coping and ultimately outcomes. Hospitalisation of their child presents a unique set of pressures and challenges for parents, but also an opportunity to intervene. However, the evidence is not well described in relation to this group of families. The primary objective of this scoping review is to identify parent and family based interventions available to improve parental health, wellbeing, functioning or skills in the context of a child's medically complex hospital admission and hospital care.

Methods and analysis: Nine bibliographic databases will be searched spanning medicine, nursing, psychology, education, social work and the grey literature using a combination of index terms and text words related to parents, childhood, chronic illness and interventions. Study eligibility will be assessed by two researchers against pre-set inclusion and exclusion criteria. Key information from each study will be extracted and charted including year of publication, condition, severity, geographical setting, key concepts and definitions, aims, study population and sample size, methodology / methods, interventions, outcomes, and key findings. Directed qualitative content analysis will be used to make sense of narrative findings within the included studies. Results will be presented which summarise the scope of the literature and identify key findings, potential areas for evidence synthesis and research gaps.

Ethics and dissemination: Ethical approval is not required. The results of this review will be disseminated through publication in a peer-reviewed journal and feedback to stakeholders during the development of a hospital based intervention.

Abstract word count: 300

Strengths and limitations of this study

- This review will describe the literature in relation to a key opportunity to intervene to improve outcomes
- This review will be based on a robust definition of children with medical complexity
- Formal scoping review methodology will be used to provide a systematic, rigorous, transparent and reproducible review

For peer review only - http://bmjopen.bmj.com/site/about/guidelines.xhtml

For peer review only

- Whilst not a systematic review this scoping review will useful for identifying focussed areas for systematic review

Background

This scoping review has been designed to inform development of an intervention to support parents of children with medical complexity around the time of hospital admission. It has been estimated that whilst children with the most complex medical needs include less than 1% of the child population they account for one third of all child health spending.[1] The impact of suboptimal management of this group of children, which can include the support given to enable successful family adjustment as well as medical management, can have a considerable effect on families as well as child health services and budgets.[2]

There is a well-established definition for children with special health care needs (CSHCN) which encompasses those children who have or are at increased risk of a chronic physical, developmental, behavioural, or emotional condition and require health care and related services of a type or amount beyond that required by children generally.[3] Definitions for groups of children with the most severe chronic diseases or diseases with the most serious long-term effects are less well established. We have adopted the definition of 'children with medical complexity' developed by Cohen [2] which is based on a systematic review of definitions of childhood chronic conditions.[4] Cohen's definitional framework includes four domains:

- Substantial family identified service needs and / or significant impact on the family (e.g. financial burden)
- Diagnosed or undiagnosed chronic condition which is severe or associated with medical fragility
- Severe functional limitations and / or dependence on technology
- High health care use and / or engagement with multiple service providers that may include non-medical providers.

When describing the wider population of children with any chronic health condition we will use the term 'children with special health care needs (CSHCN)'.[3] When referencing source literature, the original terminology will be used—e.g. chronic conditions—in order to retain a sense of the original meaning.

Most parents adjust to their child's illness successfully. [5 6] However not all families do adjust well and poor adjustment has been associated with poorer health outcomes for parents, the ill child and other family members. [7] A recent meta-analysis of 37 studies where the relationship between family

For peer review only

For peer review only - http://bmjopen.bmj.com/site/about/guidelines.xhtml

functioning and child wellbeing in children with chronic health conditions were analysed found significant correlations between family functioning and children's problem behaviours, social competence, quality of life, medication adherence and physical health. [8]

Whilst some families appear to cope more easily than others there are compelling reasons to suggest that effective interventions may improve outcomes for parents and their families. Some factors that predict adjustment may not be particularly open to hospital based intervention such as family environment, illness severity and chronicity (the long-term nature of the diagnosis). [9] However other factors that have been identified as facilitators of successful adjustment are more amenable to hospital based intervention. These include focussing on the child's achievements; performing care routines; becoming flexible in relation to care and treatment routines; developing knowledge of the condition and treatments; being able to learn from illness episodes and apply that learning to future situations; and developing effective relationships with staff. [5]

Several authors highlight the importance of the illness trajectory. Burden [10] suggests that there are opportunities for professionals to support parents to successfully adjust to their child's diagnosis. Rolland and Walsh identify three major phases of childhood chronic disease: crisis (pre-diagnosis and initial adjustment); chronic (the long haul); and terminal phases in progressive conditions. [11] These phases pose distinct challenges and are likely to be associated with healthcare contact and opportunities for supportive interventions to promote resilience and adjustment. The potential benefits of parenting programmes are highlighted in the findings of two reviews. A Cochrane review of group-based parenting interventions to improve parental psycho-social health found evidence to support the use of parenting programmes [12] and a separate Cochrane review found some evidence that psychological therapies are beneficial for parents of children with special health care need.[13] Further evidence covering related issues have also been reviewed, for example research on improving or supporting professional-parent collaborations in managing children with special health care needs,[5 14] nursing research on parenting children with complex chronic conditions,[15] the nature of family engagement in interventions for this population,[16] and the role of interactive media for parental education.[17]

However, whilst these reviews provide valuable insights they do not provide a comprehensive evidence base for the context of children with medical complexity around the point of hospitalisation. Much of the available review evidence only addresses pre-determined categories of interventions, (e.g. group [10], psychological [11], media [15]), and do not address other potentially important parent and family support functions such as social support, chronic illness education and skill development, or support with relevant common parenting issues. In addition, they are not

For peer review only - http://bmjopen.bmj.com/site/about/guidelines.xhtml

always well tailored to the specific parenting challenges around children with medical complexity. This review will address this knowledge gap by scoping a broad range of parent and family-based interventions that have been tested within populations of children with special health care needs (CSHCN). This is important because to our knowledge the wide range of evidence which could be relevant to the parents of medically complex children has not yet been scoped. This broad scoping review will allow that evidence to be identified, characterised, and assessed in relation to the needs of these parents and families during hospital admissions and in the context of hospital care.

For peer review only

Methods / design

Research question and objectives

The research questions for this review are (1) What interventions are available to improve health, wellbeing, functioning or skills in parents of children with special health care needs (CSHCN),? (2) Who are the study populations, what were the intervention targets, which outcomes have been measured, and is there evidence of efficacy or comparative effectiveness? (3) To what extent are the results relevant and transferable to delivery within routine care in a hospital setting? A further objective is to identify potential areas for full systematic review.

Study design

Scoping review methodology is particularly well suited to this research because meeting the objectives depends on identifying and summarising a broad range of potential intervention types and research methodologies. This approach also provides a rigorous, transparent and reproducible method for scoping a research area that includes a systematic search strategy and data extraction. Formal scoping review methodology will be used [18-22], drawing on Arskey and O'Malley's methodological framework[19] informed by recent Joanna Briggs Institute Guidance[18]. This includes identifying a research question, identifying relevant studies, study selection, charting the data, and collating, summarising and reporting the results.

In order to include and describe the full extent of relevant literature scoping reviews do not typically exclude studies based on design or quality, and data quality can therefore vary widely. The broad nature of many scoping reviews can also make study synthesis more problematic than in a full systematic review. However both of these limitations do allow the full extent of the relevant literature to be included and described, which is useful where an area is complex or has not been comprehensively reviewed before[19] and have been addressed in this protocol.

For peer review only - http://bmjopen.bmj.com/site/about/guidelines.xhtml

Eligibility criteria

The Population, Intervention, Comparator, Outcome (PICO) framework has been used to define the review focus and a PICO statement can be found in Table 1.

Table 1: PICO Statement

Population	Parent of CSHCN
Intervention	Any parent or family based intervention
Comparator	Usual care or any other comparator
Outcome	Improved parenting health, wellbeing, functioning or skills

Detailed study eligibility criteria can be found in Table 2.

Table 2: Inclusion and Exclusion Criteria

**Inclusion criteria**	
Types of studies	Any reports of interventions using a recognised study design (including primary or secondary research).<br><br>Interventions must aim to improve health and wellbeing, functioning or skills in parents of children with special health care needs.
Setting	Studies undertaken in any research setting (e.g. acute, primary care, community) will be included, as long as the intervention could potentially be delivered within routine care in an acute setting.
Population	'Parent' may include anyone with parenting responsibility.<br><br>Children with special health care needs (CSHCN): children who have or are at increased risk of a chronic physical, developmental, behavioural, or emotional condition and require health care and related services of a type or amount beyond that required by children generally.
Intervention	Interventions must include parents directly. They may include only parents, or

For peer review only - http://bmjopen.bmj.com/site/about/guidelines.xhtml

For peer review only

	parents alongside children and / or other family members.<br><br>Interventions may include but are not limited to peer-support, listening and encouraging, education, training, enablement, modelling, or environmental restructuring (for example care environments). Single disease studies will be included (for example cardiac conditions, cancer, metabolic conditions) as long as they meet the above inclusion criteria, as well as studies that include parents of children with a variety of clinical conditions.
Outcomes	Improved parent-child attachment or parenting health, wellbeing, functioning or skills.<br><br>'Health and wellbeing' may include patient reported outcome measures (PROMs), happiness, psychological adjustment or adaptation, quality of life, resilience, coping or self-efficacy. It may also include reduction in negative outcomes including stress, anxiety, depression, or physical health measures.<br><br>Parental functioning and skills refers to a range of parenting behaviours including nurturing, discipline, teaching, monitoring, and management. [23]

Exclusion criteria

- Any studies which do not report parent outcomes
- Interventions that are not adaptable to delivery by generalist healthcare staff or lay workers (e.g. specialist psychotherapy techniques)
- Studies which use parent- based interventions but only measure child wellbeing or disease related outcomes such as medication adherence
- Studies that focus on acute conditions only (e.g. acute pneumonia)
- Studies that focus only on end of life care
- Studies on Attention Deficit Hyperactivity Disorder (ADHD), autism, depression or other mental health conditions in the absence of co-morbidities.

For peer review only

Search strategy

A comprehensive search strategy will be developed to identify both published and unpublished literature. It will be designed and will be performed with advice and support from a specialist in systematic reviews. A range of sources will be searched including the following disciplines: medicine, nursing, allied health professions, sociology, psychology, education and social work. Peer-reviewed, published literature will be searched as well as grey literature. Grey literature will be searched in order to increase the chance of finding evaluations that not have been published in peer-review journals. Primary research studies that evaluate interventions using any methodology and secondary research studies including scoping reviews, systematic reviews and meta-analyses will be included.

Relevant studies will be identified through individual searches of relevant data bases. These will include Medline, Embase, PsycINFO, the Cochrane library, the Cumulative Index of Nursing and Allied Health Literature (CINAHL), Education Resources Information Centre (ERIC), and Applied Social Sciences Index and Abstracts (ASSIA). HMIC and OpenGrey will be searched for grey literature. Reference lists will be mined for additional references. No previous similar reviews have been found and therefore no date restrictions will be applied. Searches will be restricted to English language papers.

A phased search strategy will be used and the initial search of Medline and CINAHL will be performed using the text words shown in Table 3 and related index terms.

Table 3 – Key word search terms

Key concept	Key words
Parents	parent, mother, father, carer, guardian
Child	child, school child, kid, toddler, teen, boy, girl, minor, underage, juvenile, youth, puberty, pubescent, prepubescent, pediatric, paediatric , school, adolescent
Chronic childhood disease	Chronic, long term, activity limiting, disease, illness, disorder, condition, sickness, pain
Interventions to improve wellbeing	Intervention, therapy, trial, review, meta-analysis

The primary researcher will screen initial search results, abstracts of relevant studies will be retrieved, and will be analysed by the same researcher for text-words contained in the titles and abstracts, as well as index terms used to describe the articles. In discussion with a systematic review

For peer review only - http://bmjopen.bmj.com/site/about/guidelines.xhtml

specialist, the results from these first stage searches will be used to optimise the search strategy for second stage searching. The second stage search will be performed individually across all databases using all identified text words and index terms found in phase 1, with search terms and strategies optimised for each database.

Study selection

EndNote (Thomson Reuters, New York) will be used to manage the records identified from the literature search and to record decisions during the study selection process. Two researchers will screen all titles from the full search results and a third researcher will take a final decision where disagreements cannot be resolved. Full texts of all potentially relevant studies will then be retrieved in full and assessed by two researchers for a final inclusion decision. Finally, reference list mining will be used to identify any further eligible studies. The selection process will be illustrated using a Preferred Reporting Items for Systematic Reviews and Meta-Analyses flow diagram.

Data extraction, analysis and synthesis

One researcher will extract data using a pre-specified data extraction form which will reflect the research questions, and this will be checked by a second researcher. Key information from each included study will be charted in a table which will include the author, year of publication, medical condition(s), severity, geographical setting, academic / professional discipline, key concepts and definitions, aims, study population and sample size, study design, methodology / methods, intervention, outcomes, and key findings related to the research questions. This list is indicative only and the charting process will be iterative. As the reviewers become familiar with the evidence, the data extraction form may be updated with other headings to ensure that all relevant information is included. In addition, the risk of bias in controlled intervention studies which contain comparative information on effectiveness will be appraised using conventional systematic review methods. [24]

Directed qualitative content analysis [25] will be undertaken to analyse narrative data. Primary coding will be based on the TiDieR Framework[26] to identify author descriptions of why, what, who, how, where, when and how much, tailoring, modification and how well interventions were delivered. In terms of 'what' interventions will be coded to reflect their primary mechanism (e.g. educational, psychological) and will be further coded to reflect their theoretical underpinning. Where possible more specific codes will be applied e.g. psychological interventions will be coded to reflect whether they are behavioural, cognitive or psychodynamic etc. Data that does not fit within this approach will be identified and analysed to determine whether they represent a new coding category or a subcategory of an existing code.

For peer review only - http://bmjopen.bmj.com/site/about/guidelines.xhtml

Presentation of results, discussion and conclusions

Results will be presented visually and descriptively. Key data will be presented in tables, including a main table of all interventions that meet the inclusion criteria. Additional data tables will summarise other key features including research methodology and design, study dates, medical condition(s), severity, geographical location, academic/ professional origin and intervention function. Results of the directed qualitative content analysis will accompany the tables to further explore and discuss key findings in relation to the scoping review questions and their implications. The discussion and conclusions will also address potential areas for evidence synthesis and any identified research gaps.

Protocol amendments

Any important amendments to this protocol will be reported with the results of this review.

What this study will add

This study will describe the evidence base available for parenting interventions for parents of children with special health care need, and support development of interventions for children with medical complexity. This scoping review will contribute to a novel parent support intervention that can be delivered from within the hospital setting.

Ethics and dissemination

Scoping reviews do not require ethical approval. This protocol has not been prospectively registered on the PROSPERO database because scoping reviews are currently excluded. The results of this review will be disseminated through publication in a peer-reviewed journal and feedback to stakeholders during the development of the proposed hospital based intervention.

Authors' contributions

SB conceived of and wrote the protocol and is the guarantor for the review. CC and KS provided academic supervision and contributed to the final draft. DB provided detailed technical advice and contributed to the final draft.

Funding statement

Sally Bradshaw is funded by the NIHR CLAHRC West Midlands initiative. This paper presents independent research and the views expressed are those of the author(s) and not necessarily those of the NHS, the NIHR or the Department of Health.

For peer review only - http://bmjopen.bmj.com/site/about/guidelines.xhtml

Competing interests

None

References

1. Cohen E, Berry JG, Camacho X, et al. Patterns and costs of health care use of children with medical complexity. *Pediatrics* 2012;130(6):e1463-70. doi: 10.1542/peds.2012-0175
2. Cohen E, Kuo DZ, Agrawal R, et al. Children with medical complexity: an emerging population for clinical and research initiatives. *Pediatrics* 2011;127(3):529-38. doi: 10.1542/peds.2010-0910
3. McPherson M, Arango P, Fox H, et al. A New Definition of Children With Special Health Care Needs. *Pediatrics* 1998;102:137-40.
4. van der Lee J, Mokkink L, Grootenhuis M, et al. Definitions and Measurement of Chronic Health Conditions in Childhood A Systematic Review. *Journal of the American Medical Association* 2007;297(24):2741-51.
5. Smith J, Swallow V, Coyne I. Involving parents in managing their child's long-term condition-a concept synthesis of family-centered care and partnership-in-care. *J Pediatr Nurs* 2015;30(1):143-59. doi: 10.1016/j.pedn.2014.10.014
6. Pinquart M. Do the parent-child relationship and parenting behaviors differ between families with a child with and without chronic illness? A meta-analysis. *J Pediatr Psychol* 2013;38(7):708-21. doi: 10.1093/jpepsy/jst020
7. Cousino MK, Hazen RA. Parenting stress among caregivers of children with chronic illness: a systematic review. *J Pediatr Psychol* 2013;38(8):809-28. doi: 10.1093/jpepsy/jst049
8. Leeman J, Crandell JL, Lee A, et al. Family Functioning and the Well-Being of Children With Chronic Conditions: A Meta-Analysis. *Research in Nursing & Health* 2016;39(4):229-43. doi: 10.1002/nur.21725
9. Morawska A, Calam R, Fraser J. Parenting interventions for childhood chronic illness: a review and recommendations for intervention design and delivery. *J Child Health Care* 2014;19(1):5-17. doi: 10.1177/1367493513496664
10. Burden R. Psycho-social transitions in the lives of parents of children with handicapping conditions. *Counselling Psychology Quarterly* 1991;4(4):331-43. doi: 10.1080/09515079108254441
11. Rolland JS, Walsh F. Facilitating family resilience with childhood illness and disability. *Current Opinion in Pediatrics* 2006;18:527-38.
12. Barlow J, Smailagic N, Huband N, et al. Group-based parent training programmes for improving parental psychosocial health (Review). *The Cochrane Library* 2012;6(Supp 2):1-205.
13. Eccleston C, Fisher E, Law E, et al. Psychological interventions for parents of children and adolescents with chronic illness. 2015 doi: 10.1002/14651858.CD009660.pub3
14. Nightingale R, Friedl S, Swallow V. Parents' learning needs and preferences when sharing management of their child's long-term/chronic condition: A systematic review. *Patient Educ Couns* 2015;98(11):1329-38. doi: 10.1016/j.pec.2015.05.002
15. Rehm RS. Nursing's contribution to research about parenting children with complex chronic conditions: an integrative review, 2002 to 2012. *Nursing outlook* 2013;61(5):266-90.
16. Knafl KA, Havill NL, Leeman J, et al. The Nature of Family Engagement in Interventions for Children With Chronic Conditions. *West J Nurs Res* 2016 doi: 10.1177/0193945916664700
17. Annaim A, Lassiter M, Viera AJ, et al. Interactive media for parental education on managing children chronic condition: a systematic review of the literature. *BMC Pediatrics* 2015;15:1-13. doi: 10.1186/s12887-015-0517-2
18. JBI. Joanna Briggs Institute Reviewers' Manual: 2015 edition/supplement. Adelaide: Joanna Briggs Institute, 2015.

For peer review only - http://bmjopen.bmj.com/site/about/guidelines.xhtml

19. Arksey H, O'Malley L. Scoping studies: towards a methodological framework. *International Journal of Social Research Methodology* 2005;8(1):19-32. doi: 10.1080/1364557032000119616
20. Davis K, Drey N, Gould D. What are scoping studies? A review of the nursing literature. *Int J Nurs Stud* 2009;46(10):1386-400. doi: 10.1016/j.ijnurstu.2009.02.010
21. Levac D, Colquhoun HL, O'Brien BC. Scoping studies: advancing the methodology. *Implement Science* 2010;5:69.
22. Colquhoun HL, Levac D, O'Brien KK, et al. Scoping reviews: time for clarity in definition, methods, and reporting. *J Clin Epidemiol* 2014;67(12):1291-4. doi: 10.1016/j.jclinepi.2014.03.013
23. National Center for Parent Family and Community Engagement. Compendium of Parenting Interventions. Washington DC: Boston Children's Hospital, 2015.
24. Higgins JPT, Green S, (editors). Cochrane Handbook for Systematic Reviews of Interventions Version 5.1.0 [updated March 2011]. The Cochrane Collaboration, 2011: Available from www.cochrane-handbook.org. 2011.
25. Hsieh HF, Shannon SE. Three approaches to qualitative content analysis. *Qual Health Res* 2005;15(9):1277-88. doi: 10.1177/1049732305276687
26. Hoffmann TC, Glasziou PP, Boutron I, et al. Better reporting of interventions: template for intervention description and replication (TIDieR) checklist and guide. *BMJ* 2014;348:g1687. doi: 10.1136/bmj.g1687

For peer review only

PRISMA-P (Preferred Reporting Items for Systematic review and Meta-Analysis Protocols) 2015 checklist: recommended items to address in a systematic review protocol*

Manuscript ID bmjopen-2016-015242

For peer review only

Section and topic	Item No	Checklist item	Location and comments
**ADMINISTRATIVE INFORMATION**			
Title:			
Identification	1a	Identify the report as a protocol of a systematic review	Title - Identified as a scoping review
Update	1b	If the protocol is for an update of a previous systematic review, identify as such	N/A
Registration	2	If registered, provide the name of the registry (such as PROSPERO) and registration number	N/A
Authors:			
Contact	3a	Provide name, institutional affiliation, e-mail address of all protocol authors; provide physical mailing address of corresponding author	Page 1
Contributions	3b	Describe contributions of protocol authors and identify the guarantor of the review	Page 10
Amendments	4	If the protocol represents an amendment of a previously completed or published protocol, identify as such and list changes; otherwise, state plan for documenting important protocol amendments	Page 10
Support:			
Sources	5a	Indicate sources of financial or other support for the review	Page 10
Sponsor	5b	Provide name for the review funder and/or sponsor	Page 10
Role of sponsor or funder	5c	Describe roles of funder(s), sponsor(s), and/or institution(s), if any, in developing the protocol	N/A
**INTRODUCTION**			
Rationale	6	Describe the rationale for the review in the context of what is already known	Pages 3-5
Objectives	7	Provide an explicit statement of the question(s) the review will address with reference to participants, interventions, comparators, and outcomes (PICO)	Page 5 and 6
**METHODS**			
Eligibility criteria	8	Specify the study characteristics (such as PICO, study design, setting, time frame) and report characteristics (such as years considered, language, publication status) to be used as criteria for eligibility for the review	Page 6

Information sources	9	Describe all intended information sources (such as electronic databases, contact with study authors, trial registers or other grey literature sources) with planned dates of coverage	Page 7-8
Search strategy	10	Present draft of search strategy to be used for at least one electronic database, including planned limits, such that it could be repeated	Page 8
Study records:			
Data management	11a	Describe the mechanism(s) that will be used to manage records and data throughout the review	Page 9
Selection process	11b	State the process that will be used for selecting studies (such as two independent reviewers) through each phase of the review (that is, screening, eligibility and inclusion in meta-analysis)	Page 9
Data collection process	11c	Describe planned method of extracting data from reports (such as piloting forms, done independently, in duplicate), any processes for obtaining and confirming data from investigators	Page 9
Data items	12	List and define all variables for which data will be sought (such as PICO items, funding sources), any pre-planned data assumptions and simplifications	Page 9
Outcomes and prioritization	13	List and define all outcomes for which data will be sought, including prioritization of main and additional outcomes, with rationale	Page 9
Risk of bias in individual studies	14	Describe anticipated methods for assessing risk of bias of individual studies, including whether this will be done at the outcome or study level, or both; state how this information will be used in data synthesis	Page 9
Data synthesis	15a	Describe criteria under which study data will be quantitatively synthesised	N/A
	15b	If data are appropriate for quantitative synthesis, describe planned summary measures, methods of handling data and methods of combining data from studies, including any planned exploration of consistency (such as I^2, Kendall's τ)	N/A
	15c	Describe any proposed additional analyses (such as sensitivity or subgroup analyses, meta-regression)	N/A
	15d	If quantitative synthesis is not appropriate, describe the type of summary planned	Page 9
Meta-bias(es)	16	Specify any planned assessment of meta-bias(es) (such as publication bias across studies, selective reporting within studies)	N/A
Confidence in cumulative evidence	17	Describe how the strength of the body of evidence will be assessed (such as GRADE)	Page 9

*** It is strongly recommended that this checklist be read in conjunction with the PRISMA-P Explanation and Elaboration (cite when available) for important clarification on the items. Amendments to a review protocol should be tracked and dated.** **The copyright for PRISMA-P (including checklist) is held by the PRISMA-P Group and is distributed under a Creative Commons Attribution Licence 4.0.**

From: Shamseer L, Moher D, Clarke M, Ghersi D, Liberati A, Petticrew M, Shekelle P, Stewart L, PRISMA-P Group. Preferred reporting items for systematic review and meta-analysis protocols (PRISMA-P) 2015: elaboration and explanation. BMJ. 2015 Jan 2;349(jan02 1):g7647.

For peer review only
